# The Roles of Uterine Natural Killer (NK) Cells and KIR/HLA-C Combination in the Development of Preeclampsia: A Systematic Review

**DOI:** 10.1155/2020/4808072

**Published:** 2020-03-28

**Authors:** Xiuhua Yang, Yahui Yang, Yiru Yuan, Lin Liu, Tao Meng

**Affiliations:** ^1^Department of Obstetrics, The First Hospital of China Medical University, Shenyang, Liaoning, China; ^2^China Medical University, Shenyang, Liaoning, China

## Abstract

Preeclampsia (PE) is termed as a systemic disease that involves multiple organs; however, the exact etiology is still quite unclear. It is believed that the poor remodeling of uterine spiral arteries triggers PE, thereby causing failed placentation and producing inflammatory factors. The decline of blood flow results in lowering the nutrients and oxygen received by the fetus and augmenting the placental pressure in PE. Decidual immune cells, especially uterine natural killer (uNK) cells, are involved in the process of placentation. Decidual NK (dNK) cells significantly contribute to the vascular remodeling through the secretion of cytokines and angiogenic mediators in normal placental development. The abnormal activation of NK cells in both the peripheral blood and the decidua was counted among the causes leading to PE. The correlation existing between maternal killer cell immunoglobulin-like receptor (KIR) and HLA-C in trophoblast cells constitutes a robust evidence for the genetic etiology of PE. The combinations of the two kinds of gene systems, together with the KIR genotype in the mother and the HLA-C group in her fetus, are likely to exactly decide the pregnancy outcome. The women, who have the inappropriate match of KIR/HLA-C, are likely to be prone to the augmented risk of PE. However, the combinations of KIR/HLA-C in PE undergo ethnic changes. The extensive prospective research works in Europe, Asia, and Africa are required for providing more findings in PE patients.

## 1. Introduction

Preeclampsia (PE) refers to quite a serious obstetrical complication that has high blood pressure and proteinuria, occurring following the 20-week period of pregnancy, and it threatens the life of both the mother and the neonate. In accordance with the statistics of World Health Organization (WHO), one-tenth of the pregnant females suffer from PE, and PE constitutes one-seventh of the deaths in pregnant women [1, 2]. The occurrence of PE in China amounts to 5% [3]. PE is termed as a systemic disease that involves multiple organs including the nervous system, blood system, heart, liver, and kidney [4]. In case of the ineffective control of the symptoms, PE is expected to develop into convulsion or coma, termed as eclampsia. Moreover, severe PE is likely to cause fetal growth restriction (FGR) or even fetal death owing to the placental vascular dysplasia. In treating PE, magnesium sulfate is usually put to use for the purpose of preventing eclampsia [5]. In addition, if systolic blood pressure amounts to higher than 160 mmHg or diastolic blood pressure is above 110 mmHg, antihypertensive drugs are usually put to use intravenously, such as labetalol [6]. Angiotensin-converting enzyme (ACE) inhibitors cannot be utilized in pregnancy owing to their teratogenic function on the neonate [7]. Owing to the fact that the current treatment is incapable of effectively alleviating the symptoms of PE, we require further exploring the pathogenesis of this disease, aimed at finding a better treatment.

Even though a number of factors have been discovered as correlated with the occurrence of PE, the exact etiology is still quite unclear. These causes count on not only environmental factors but also immunological factors, genetic factors, vascular endothelial cell damage, blood system abnormalities, and some unidentified factors [8–10]. In PE, trophoblast cells fail in invading optimally [11]. It is believed that the poor remodeling of uterine spiral arteries triggers PE, thereby causing the failed placentation and producing inflammatory factors. PE patients have immune inflammation as well as the generation of autoimmune antibodies [12]. Inflammatory mediators result in the activation of maternal endothelial cells, which have the potential of causing hypertension and proteinuria [13, 14]. In the present review, we provided the summary of the roles of uterine natural killer (NK) cells and killer cell immunoglobulin-like receptor (KIR)/HLA-C combination in the development of PE according to the literature published in the past few years. Also, the current manuscript aims at identifying the theoretical basis for the treatment of immune inflammation in PE, together with improving the outcome for the neonates and the women having PE.

## 2. Reduced Blood Flow during Placentation in PE

In the early phase of normal pregnancy, the uterine arteries undergo changes in the structure, thereby increasing the blood flow to the uterus by 100 times [15]. The transformation of uterine arteries has a close correlation with placentation. In the process of placentation, fetal trophoblasts from the placenta immerse into the uterine wall, besides implanting into uterine arteries and penetrating the smooth muscle of the uterus. This change in trophoblasts makes uterine arteries significantly conductive catheters, leading to the decline of the speed and pressure of uterine blood flow into the placenta. The cessation of uterine artery dilation further lowers the velocity of blood flow into the villous space. This provides sufficient time for exchanging the nutrients between the mother and the fetus, in particular, when the demand for nutrients is the highest in the late pregnancy. In addition, some important signaling pathways including YY1/MMP2 play important roles in the invasion of trophoblasts during the first trimester [16].

**Figure 1:**
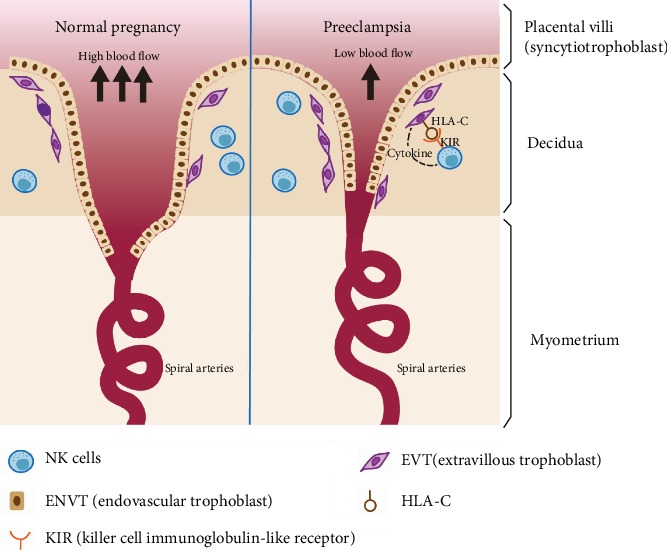
Preeclampsia (PE) is related to the poor placentation in the early pregnancy. In normal early pregnancy (left picture), extravillous trophoblast cells (EVT) invade deeply enough in the myometrium and also migrate into the endothelium of maternal spiral arteries. This ensures that there is abundant blood flow at the maternal fetal interface. However, in PE patients (right picture), the depth of trophoblast invasion is decreased with insufficient remodeling of trophoblast cells. Blood flow is also reduced in PE. Inappropriate combination of KIR/HLA-C in PE will inhibit the functions of NK cells including secreting angiogenic cytokines. As a result, uterine NK (uNK) cells in these women have low functional activity and they do not support placental growth as needed.

In PE, trophoblast cells fail in helping with the structural transformation of arteries, thereby causing the artery blood to flow into the villus space without essential conversion; also, it causes the injury of the villus structure. The decline of blood flow results in lowering the nutrients and oxygen received by the fetus and augmenting the placental pressure [17]. Accordingly, one of the main causes of PE is the insufficient remodeling of uterine arteries [18]. Decidual natural killer (dNK) cells and extravillous trophoblasts (EVT) are involved in placental formation [19]. Now, a number of scholars hold the belief that the unusual immune response of the mother to the fetus constitutes a preliminary factor of PE, which causes the systemic inflammatory response in the female [20]. A number of evidence suggest that PE is a result of poor placentation in early pregnancy [21, 22] (Figure 1).

## 3. The Process of Placentation Involved by Immune Cells in PE

For the purpose of comprehending the mechanism of the decidua regulating placentation, the decidual immune cells have been concentrated on [23, 24]. Considering that the reason of immune cells is from the epidemiological investigation of PE [25], firstly, it refers to a disease, occurring in the first pregnancy, after which the mother could get immunity. Changing the father following a normal pregnancy is likely to induce PE; nonetheless, if the patients, having had PE change their sexual partners, the incidence of PE is going to be lowered [26]. Moreover, the incidence of this disease has memory and specificity, which is consistent with the characteristics of immune diseases. There have been a number of investigations dealing with the family history and genetics of mothers; furthermore, several research works have shed light on the fact that the paternal factor also plays a major role in the incidence of PE, together with its association with the fetal weight [27–29]. Numerous research works have revealed that the relationship between the maternal and fetal immune systems has the potential of determining the outcome of pregnancy. The immune cells in the decidua play quite a critical role at the maternal fetal interface. Since the mother and the fetus form the two different genetic individuals, the invading trophoblasts carry genes and molecules with the paternal source; in immunologic terms, the fetus is alien to the mother.

The hypothesis that decidual immune cells are involved in the placentation is primarily owing to two reasons. Firstly, the cell-cell interaction in the decidua takes place between the two allogeneic individuals. Secondly, the pivotal role of the decidua in placentation is reflected in the investigation of obstetrical complications. In the patients having placenta percreta with the absence of the decidua, the trophoblasts deeply invade the uterine muscle wall. In this event, the placenta is most likely to grow in the scar of the former cesarean section [30]. In the early pregnancy, 70% of neutrophils in the endometrium are uNK cells. These cells have KIRs, combining with HLA-C ligands in the trophoblasts [31]. Owing to the genetic variability of KIR as well as HLA-C, there are a number of varying types of combination of not only maternal KIR but also fetal HLA-C in each of the pregnancies [32]. Moreover, integrating the KIR and HLA-C figures out whether uNK cells are capable of secreting angiogenic cytokines. This field is comparatively newer; nonetheless, the comprehension of this knowledge could offer new perceptions and ideas not only for the diagnosis but also for the treatment of obstetrical complications like PE.

## 4. uNK Cells in the Pathogenesis of PE

Which type of immune cells is likely to be involved in the development of PE? Our answer is uNK cells, because they account for the majority (70%) of leukocytes in the process of implantation and placentation, and they have receptors that could combine with ligands in the trophoblasts. In spite of T cells, as the effector immune cells in charge of rejecting organ transplants, which account for 10 to 30% of leukocytes in the endometrium in the early phase of pregnancy, no available investigation indicates that the failure of pregnancy is a result of the rejection of T cells to the placental tissue [33]. Precisely, there are no research works that have found that maternal T cells are capable of recognizing and acting on trophoblasts. Approximately 90% of pNK cells are cytotoxic, together with having a CD56^dim^CD16^+^ surface phenotype, and the remaining 10% are CD56^bright^CD16^−^ phenotypes with little cytotoxicity [24, 34]. In addition, immune factors were collaborative for characterizing the pregnancy as a mildly inflammatory condition. The proportion of pNK cells undergoes a gradual increase in the early phase of pregnancy, together with a decrease in the middle phase of pregnancy, continuing the decline in the third trimester in a normal pregnancy [35]. Carolis et al. were of the belief that the changes in pNK cells played a pathogenic role in PE [36]. The abnormal activation of NK cells in both the peripheral blood and the decidua was counted among the causes leading to PE [36].

The uNK cells differ with pNK cells in phenotype and function [17]. uNK cells are phenotypic CD16^−^CD56^bright^ NK cells with little cytotoxicity that have a direct contact with the allogeneic EVT cells. uNK cells are regarded as playing a pivotal function in the adjustment of fetal EVT for the establishment of a fine placentation [35]. The specific uNK cells (CD56^+^, CD3^−^, CD16^−^, and CD9^+^) were similar in the late and early pregnancies, which demonstrated that these uNK cells contributed to the normal development of the fetus all through the entire pregnancy [37]. Mice without uNK cells do not have the compatible vascular formations associated with pregnancy [38, 39].

There are two different kinds of uNK cells that have been confirmed in mice in accordance with their activities towards Dolichos biflorus agglutinin (DBA) [40]. DBA^+^ uNK cells produce angiogenic mediators, while DBA^−^ uNK cells secrete IFN-*γ* [40]. There was an experiment that had a more rigorous design, making use of the alymphoid mice, achieving the bone marrow from either IFN-*γ*^−/−^ mice or serious combined immunodeficient mice, which were absent for T and B lymphocytes; thereafter, they discovered the fact that the IFN-*γ* produced by NK cells was quite pivotal for the spiral artery remodeling [41]. In another research work, the researchers made use of BPH/5 mice, having the core characteristics of PE; also, they discovered that there was a decline in the number of dNK cells in their decidua [42]. The reduction of uNK cells had an association with the upregulation of Cox2 and IL-15 at the uterus-placenta interface [42]. Following the addition of the Cox2 inhibitor, lowering the expressions of Cox2 and IL-15, the number of uNK cells recovered [42].

Furthermore, the invasion of trophoblast cells in mice was lower as compared with that in human beings, so trophoblast cells in mice significantly differ with those in humans [43]. Even though the majority of NK receptors in mice are from the Ly49 receptor family, their function seems to have a similarity with KIR in humans. With regard to mouse studies, on the addition of H2-Dd, the vascular remodeling was declined and fetal growth was decreased in comparison with the homotypic mice that lacked merely H2-Dd [44]. This major histocompatibility complex (MHC) molecule has the potential to bind to the inhibitory receptor Ly49A, besides decreasing the extra uNK subtype cells on their appearance [44]. Being specific, the growth rate of fetus slowed down irrespective of the parental source of the H2-Dd molecule [44]. These findings suggest that some combinations of maternal NK receptors and paternal/maternal MHC groups had the potential of impacting the trophoblast invasion and vascular remodeling. The research works dealing with the pregnant transgenic mice discovered the fact that the uterine spiral arteries of transgenic mice, which lacked uNK cells, were aberrantly straight as well as narrow [45, 46]. In mice studies, adrenomedullin (AM), a pregnancy-related peptide, has been termed as a pivotal factor, facilitating the accumulation and activation of maternal uNK cells to the placenta, together with helping the process of spiral arteries remodeling eventually [47]. The placentas that lack AM or its receptor manifested the decreased fetal vessel branching in the uterus, the failure of spiral artery remodeling, and re-endothelialization, in addition to apparently decreasing the amounts of maternal uNK cells [47].

## 5. Angiogenic Factors Produced by uNK Cells in PE

The human placenta experiences the elevated degrees of angiogenesis as well as vasculogenesis all through the growth of the fetus [48]. Also, the human placenta experiences the phase of pseudovascularization, which indicates that all through the mechanism of placentation, the cytotrophoblasts of the placenta are transformed from the epithelial type to the endometrial type [49]. PE is featured by the extensive systemic impairment of endothelial cells in the maternal body [50]. Currently, a general belief is held that the incidence of PE is owing to the placental vascular dysplasia; contrarily, this changed placenta is expected to cause the extensive damage of vascular endothelial cells [51]. The declining reconstruction of uterine spiral arterioles is considered the outcome of the defect of the intravascular invasion as well as the damaged formation of pseudovessels [52]. Both the animal and human experiments have discovered the fact that PE takes place when the invasion of trophoblast declines, besides the occurrence of the uterine placental hypoperfusion. For instance, in animal experiments, it was discovered that placental ischemia caused the continuous mechanical contraction of uterine arteries and aorta, thereby causing hypertension, proteinuria, and endothelial hyperplasia of renal tubules [53]. Besides that, the pathological report of severe PE patients sheds light on the fact that the placenta has infarction and the arteries have rigid stenosis [54]. By means of ultrasonic monitoring, it could be discovered that, prior to the medical manifestations emerging in PE patients, the blood flow between the uterus and the placenta undergoes a decline, coupled with the increase in the resistance of uterine blood vessels [55]. Nonetheless, this change was observed as insignificant in one-third of PE patients. The placental ischemia itself is deemed as insufficient for causing PE. A number of factors, promoting or inhibiting the angiogenesis, significantly contribute to the placental development [56].

As indicated by in vitro experiments, dNK cells could secrete two cytokines that include interleukin-8 (IL-8) and interferon-inducible protein-10 (IP-10), promoting the invasion of trophoblast cells [54]. Subsequent to the addition of the monoclonal antibodies of IL-8 as well as IP-10 to the cultured trophoblast cells, the migration capability of cells underwent a decline [54]. In vitro, dNK cells also secreted factors promoting angiogenesis, for instance, vascular endothelial growth factor (VEGF) and placental growth factor (PlGF) [57]. In comparison with peripheral NK (pNK) cells, the secretion of VEGF and PlGF augmented significantly following the addition of IL-15 in dNK cells [57]. The migration of human umbilical vascular endothelial cells (HUVEC) was augmented in dNK cells supplied with IL-15 in vitro, besides the reticular structure appearing earlier; nonetheless, they did not receive the same impacts in pNK cells [57]. As the researchers added Flt1-Fc, which was an inhibitor of the VEGF and PlGF signal pathway, some of these functions were declined [57]. Following the subcutaneous injection of dNK cells and JEG-3 choriocarcinoma cell line into the nude mice, the volume of JEG-3 tumor and the number of blood vessels augmented, which suggested that dNK cells had the potential of promoting angiogenesis [57]. In the earliest phase of arterial recasting, matrix metalloproteinase-7 (MMP-7) and MMP-9 were observed in dNK cells in the specimens of the decidua basalis, which suggested that dNK cells had involvement in the independent stage of arterial recasting [58]. In mouse experiments, not only TGF-*β* but also PlGF and VEGF contributed to the angiogenesis [59]. These experiments suggest that dNK cells significantly contribute to the vascular remodeling through the secretion of cytokines and angiogenic mediators in the development of the placenta.

## 6. The Roles of KIR and HLA-C in Immunity, Normal Pregnancy, and Preeclampsia

Under physiological conditions, the acting ways between KIRs and HLA class I ligands determine whether NK cells can play normal functions. The consequence of KIR and HLA combination on NK cell function could change according to the resting status or in an immune state. For instance, if there is under the resting status, inhibitory KIRs make NK cells play a functional role, while activating KIR decreases NK cell abilities when combined with their cognate ligand (called NK cell education). In an immune state, inhibitory KIR could reduce NK cell ability unless HLA class I expression is decreased, while activating KIR could prime NK cell roles. The KIR/HLA combination is very complex and extremely polymorphic. The relationships between KIRs and HLAs are related to many diseases, including infectious diseases, autoimmune diseases, malignant tumors, and transplant reactions [60–63]. In pregnancy, the inhibitory or activating KIRs are capable of regulating the activity of uNK cells, thereby playing an immunomodulatory role at the maternal fetal interface. KIR A do not have stimulatory receptors, whereas KIR B have both stimulatory and inhibitory receptors. In each of the pregnancies, the KIR genes of the pregnant woman are expected to change, since these KIR genes are inherited and expressed by uNK cells. The paternal HLA-C group is also expected to be different (even from the same father), for the reason that the fetus is likely to inherit any group of HLA-C from the father. Besides that, the mixes of the two kinds of gene systems, together with the KIR genotype in the mother and the HLA-C group in her fetus, are likely to exactly decide the reaction between trophoblast cells and uNK cells.

The correlation existing between KIR/HLA-C and PE constitutes a robust evidence for genetic etiology of PE (Table 1). Until today, the largest study that ever took place in Britain involved 200 patients, who had PE in the experimental group, together with 201 women in the control group with normal deliveries [64]. When the mother had inhibitory KIR (KIR AA genotype), besides the fetus having HLA-C2, it was more likely to have the abnormalities during the spiral artery remodeling and defective placentation, eventually resulting in PE [64]. In comparison with C1, C2 combines more closely with homologous KIR. Moreover, inhibitory KIR has KIR2DL1, capable of strongly inhibiting NK cells. Nevertheless, there is no activating KIR at this time, failing in providing activation signals. Consequently, NK cells in these women manifested low functional activity, besides not supporting placental growth as required.

Nonetheless, an extensive research from Japan did not support this finding [65]. To our understanding, Caucasian men are more likely to carry HLA-C2 allele as compared with Japanese men. Accordingly, for Japanese women, the risk of PE in combination with Caucasian men should be higher as compared with that in combination with Japanese men. However, no expected experimental results have been attained that the incidence of PE in the former combination was lower as compared with that in the latter (1.54% *vs.* 2.67%) [65].

It requires observation that the proportion of KIR AA in patients having PE augmented only when the fetus inherited paternal HLA-C2 [19]. Obstetrical complications had lower likelihood of occurrence in the females, having KIR B genotype, including activating KIR2DS1, which was bound, in particular, to HLA-C2 [19]. uNK cells produce a number of cytokines that include TGF-*β*, PlGF, and VEGF, which may be of pivotal significance in guiding immune reactions [57, 66]. KIR2DS1 is capable of stimulating uNK cells that augment the angiogenesis and immune response, thereby resulting in healthy pregnancy, whereas inhibiting uNK cells is likely to lower the secretion of cytokines, thereby causing PE [66]. It has been discovered that KIR2DS1-positive females having a fetal HLA-C2 had a preferable trophoblast invasion as well as spiral artery remodeling through the secretion of granulocyte-macrophage colony-stimulating factor (GM-CSF), while in the females having the KIR AA genotype, PE was more likely to take place [67].

The correlation between activating KIR genes and lower risk of PE changes among different populations. KIR2DS5 protectively contribute to Ugandans, which are unique to sub-Saharan Africa (SSA) [68]. Researchers are unaware of the fact of what the ligands of KIR2DS5 are; nonetheless, all of the research works carried out the single European KIR2DS5∗002 allele that refers to an activating KIR frequently observed in the tel-B in European people. Together with that, the KIR2DS5∗006 allele refers to a protective allele that appears in the cen-B in SSA and can be activated while binding to HLA-C2 [68].

It has also been illustrated that the expressions of KIR2DL/S1, 3, and 5 were decreased on the percentage of dNK cells in a case where patients had elevated uterine artery resistance index (RI), indicating poor spiral artery remodeling [65]. This is termed as the mechanical application of PE as a result of the interactions between dNK cells and trophoblasts [69].

In the context of China, women having PE have an evidently larger frequency of KIR AA genotype, primarily containing the inhibitory receptors, in addition to the lower frequency of maternal activating gene KIR2DS1 as compared with normal pregnancies [70]. Furthermore, this finding shows consistency with earlier research works in other populations [66, 67]. It is believed that activating KIR2DS1 refers to a protective determinant, and insufficient activation of uNK cells is expected to lead to decreased invasion of trophoblasts, thereby resulting in PE [70]. Moreover, it was also indicated that if the fetus possessed more numbers of HLA-C2 genes as compared with the mother, the maternal KIR AA genotype was correlated with a higher risk for PE [70]. This research work also supports the hypothesis that immune factors from fathers contribute to the development of PE [71]. In another extensive investigation from China, there were 271 women in the experimental group, together with 295 women in the control group, who were collected with the use of the polymerase chain reaction with sequence specific primers (PCR-SSP) assay [72]. They figured out that PE patients had fewer activating KIR2DS2, KIR2DS3, and KIR2DS5 [72]. Besides that, the gene frequency of total activating KIRs in PE group was evidently smaller in comparison with that of the control group (*P* = 0. 03) [72].

PE patient showed more likelihood of being KIR2DL1 positive when the fetus had HLA-C2C2; in addition, in this case, uNK cells were expected to receive the strongest inhibitory signals [72]. Furthermore, the same trend was also discovered in Mexico [37]. 10 normal decidual specimens and 9 decidual samples from PE patients were employed in the process of cesarean section [37]. They discovered that inhibitory KIRs were predominated in PE patients in comparison with normal pregnant women [37].

Besides that, it has also been highlighted that activating maternal KIR-B genotype itself, in combination with fetal HLA-C2, had an evident correlation with decidual acute atherosis in PE patients [73]. In PE patients having acute atherosis, the incidence of this combination amounted to be 60%, whereas, in PE patients not having acute atherosclerosis, the rate was 24.5% (*P* = 0.001) [73]. They held the belief that the appearance of acute atherosis was a result of decidual inflammatory reactions owing to the reactions between fetal HLA-C2 and maternal activating KIRs on dNK cells [73].

Nevertheless, some negative findings were made as well. In a Danish study, 259 pregnant females, who had severe PE or eclampsia in the trial group, together with 259 pregnant females, who did not have PE or eclampsia in the control group, were enrolled [74]. The blood of these pregnant women as well as their newborns was gathered [74]. No correlation existing between maternal KIR AA and HLA-C2 in their newborns was observed [74]. With the newborns carrying more HLA-C2 allele as compared with the pregnant women, no difference in maternal KIR AA genotype between the trial cohort and the control cohort was observed [74].

Contradictory results of KIR/HLA-C combination in PE patients are likely owing to the changes in KIR gene and repertoire frequencies between different ethnicities. KIR genotypes also have an extensive variation in geographical distribution. Therefore, the direct comparison of these studies about KIR and HLA correlation with PE is a difficult task because they were conducted in various populations, together with distinct methods.

With regard to the future studies, it is necessary to carry out large-scale prospective randomized controlled research on different ethnic groups in Europe, Asia, and Africa, and researchers should select suitable control groups for their studies, simultaneously collect KIR classification of mothers and HLA-C groups of husbands and neonates, and analyze and judge whether different combination types of KIR/HLA-C are related to the prognosis of mothers and newborns.

Except for class I HLA-C, EVT also express atypical class Ib HLA-E, F, and G [75]. HLA-G can inhibit the effect of NK cells [76]. In the first trimester, the embryo could produce soluble HLA-G [77] and it is important for immunotolerance in maternal fetal interface [78]. Compared with nonpregnant females, the expression of soluble HLA-G in serum of pregnant women at all stages was significantly higher [79] and the soluble HLA-G increased the production of IL-10 [80]. It was found that the expression of soluble HLA-G in the serum and placenta of PE women was significantly lower than that of normal pregnant women [81–85]. It is suggested that soluble HLA-G may be involved in the pathogenesis of PE. Recently, it has been found that class II HLA-DR can be detected in placentas from PE patients (*n* = 23), but not in normal placentas (*n* = 14) [86]. The mechanism of HLA-DR in PE needs to be further explored.

## 7. Conclusions

To conclude, NK cells are existent in the decidua in abundance in early pregnancy, which are of immense significance for the maintenance of normal pregnancy. In the mechanism of placentation, uNK cells require necessary activation for the purpose of releasing cytokines, promoting angiogenesis, and helping remodel uterine spiral arteries. The women, who have the inappropriate match of KIR/HLA-C, are likely to be prone to the augmented risk of PE. With regard to these women, the RI of the uterine artery could be monitored in early pregnancy, whereas timely and effective intervention could be performed for the prevention of PE.

Because of reproductive failure, more and more couples choose gestational carriers. In 2013, gestational carriers accounted for 2.5% of all assisted reproductive technologies in USA [87]. The incidence of PE in gestational carriers has not been reported, while that of multiple births and preterm birth is relatively high [88]. Accordingly, HLA-C and KIR genotyping could be potentially applicable for selecting the third party gametes or gestational carriers, aimed at avoiding the obstetrical complications including PE. In clinical work, for the high-risk patients of PE, the role of uNK cells in the process of placentation should be taken into account; for the women with high-risk combinations of KIR/HLA-C, the frequency of prenatal examination should be increased.

## Figures and Tables

**Table 1:** Studies on killer cell immunoglobulin-like receptor (KIR)/HLA-C in preeclampsia (PE) sorted by publication date.

Ethnicity	Authors	The year of publication	The experimental group	The control group	Samples	Conclusions
British	Hiby et al. [64]	2004	PE patients (*n* = 200)	Full-term pregnant women (*n* = 201)	Mothers: blood Babies: umbilical cord blood or mouth swabs	The combination of maternal KIR AA and fetal HLA-C2 was more common in PE.
Japanese	Saito et al. [65]	2006	Couples with Japanese women and Caucasian men (*n* = 328)	2003 database in Japan (*n* = 36,829)	—	There was no statistical difference in the incidence of PE between the two groups.
White British	Hiby et al. [20]	2010	PE patients (*n* = 742)	Normal primiparas (*n* = 592)	Mothers: blood Babies: umbilical cord blood or mouth swabs	Maternal KIR AA was related to PE when the fetus had more HLA-C2 inherited from the father. Maternal telomeric KIR B (KIR2DS1) was a protective factor for PE.
Mexico	Sánchez-Rodríguez et al. [37]	2011	PE patients (*n* = 9)	Normal pregnant women (*n* = 10)	Mothers: decidual samples	PE patients tended to have more inhibitory KIRs.
Chinese Han population	Yu et al. [70]	2014	PE patients (*n* = 47)	Normal pregnant women (*n* = 54)	Mothers and fathers: blood Babies: umbilical cord blood	Less PE patients had KIR2DS1, and more PE patients had AA genotype compared with normal pregnant women. More PE patients with KIR AA had fewer HLA-C2 than their babies.
Chinese Han population	Long et al. [72]	2014	PE patients (*n* = 271)	Normal pregnant women (*n* = 295)	Mothers: blood Babies: umbilical cord blood	PE patients had less activating KIRs (2DS2, 2DS3, and 2DS5). The frequency of KIR2DL1 was increased in PE patients when the neonate was HLA-C2C2.
Uganda (sub-Saharan Africans)	Nakimuli et al. [68]	2015	PE patients (*n* = 254)	Normal pregnant women (*n* = 484)	Mothers: blood Babies: umbilical cord blood	The combination of maternal KIR AA and fetal HLA-C2 was related with PE. KIR2DS5 and KIR2DL1 had the protective effect on PE.
European	Johnsen et al. [73]	2018	PE patients (*n* = 83)	Normal pregnant women (*n* = 83)	Mothers: blood, decidua, or muscle Babies: umbilical cord blood or fetal placenta	PE patients with acute atherosis tended to have the combination of maternal KIR-B and fetal HLA-C2 compared with PE patients without acute atherosis.
European	Larsen et al. [74]	2019	Severe PE patients (*n* = 259)	Normal pregnant women (*n* = 259)	Mothers: blood Babies: blood	There was no effect of KIR/HLA-C combination on the risk of severe PE.
